# Red cell distribution width and hypertension: Associations from NHANES 2021 to 2023 study and MR-PRESSO-adjusted Mendelian randomization analysis

**DOI:** 10.1097/MD.0000000000046680

**Published:** 2025-12-12

**Authors:** Zihao Zhao, Yuhong Ma, Weizhong Huangfu

**Affiliations:** a Department of General Practice, The Affiliated Hospital of Inner Mongolia Medical University, Hohhot, Inner Mongolia Autonomous Region, China.

**Keywords:** hypertension, Mendelian randomization, NHANES, nonlinear association, red cell distribution width

## Abstract

Hypertension, a common chronic disease worldwide, has an unclear causal relationship and association pattern with red cell distribution width (RDW), a prognostic marker for cardiovascular events. Traditional observational studies have struggled to clarify this relationship due to confounding factors and reverse causality. A mixed study design integrating observational and genetic evidence was adopted: observational analysis: based on data from National Health And Nutrition Examination Survey 2021 to 2023 (n = 5768), complex sampling-weighted multivariable logistic regression and restricted cubic splines were used to analyze the RDW-hypertension association, with subgroup interaction tests; causal inference: MR-PRESSO-adjusted Mendelian randomization (MR) analysis (hypertension GWAS: ukb-b-12493; RDW GWAS: ebi-a-GCST006804) was performed, using 56 strong instrumental variables (*F* > 10) to verify the causal direction, supplemented by sensitivity analyses including inverse-variance weighting (IVW) and MR-Egger. Each 1-unit increase in RDW was associated with a 13% increased risk of hypertension (OR = 1.13, 95% CI = 1.05–1.21) after adjustment for 12 covariates in the full model, with a nonlinear threshold effect (*P* for nonlinearity = .028): the association was stronger when RDW < 13.8% (OR = 1.29, *P* < .001). After MR-PRESSO adjustment, the IVW method confirmed that hypertension caused an increase in RDW (OR = 1.36, 95% CI = 1.03–1.79, *P* = .03), with no horizontal pleiotropy (Egger intercept = 0.973). Subgroup analysis showed that the RDW-hypertension association was significant in populations without a history of stroke (OR = 1.14) or coronary heart disease (OR = 1.14) but disappeared in patients with these conditions (interaction *P* < .05). This study is the first to explore the association between hypertension and RDW via MR-PRESSO-adjusted MR, providing evidence of a potential causal link where hypertension may contribute to increased RDW, and identifies a RDW threshold of 13.8%. RDW can serve as a prognostic marker for cardiovascular events and may be a reference indicator for monitoring the risk of hypertension in populations without cardiovascular complications, pending further validation of its role in the pathological mechanism of hypertension.

## 1. Introduction

Hypertension, as one of the most common chronic non-communicable diseases worldwide,^[1]^ has become the primary risk factor for the onset and death of major diseases such as cardiovascular diseases, stroke, and chronic kidney disease.^[2–4]^ According to statistics from the World Health Organization, the number of people with hypertension worldwide has exceeded 1 billion, and its threat to public health is increasingly severe.^[5]^ In clinical practice and basic research, exploring the pathophysiological mechanisms related to hypertension and potential biomarkers has always been a research hotspot in the field of cardiovascular diseases.^[6]^

Red cell distribution width (RDW) is a routine hematological index reflecting the heterogeneity of red blood cell volume, which was initially mainly used for the differential diagnosis of anemia.^[7,8]^ In recent years, more and more studies have shown that RDW is closely related to various cardiovascular diseases, including coronary heart disease, heart failure, atrial fibrillation, etc., and has been regarded as an important marker for predicting the prognosis of cardiovascular events.^[9–12]^ However, regarding the relationship between hypertension and RDW, existing epidemiological studies have suggested a correlation between the 2, but the research conclusions are controversial, and most studies have failed to clarify the causal relationship between them.^[13,14]^ Traditional observational studies are susceptible to confounding factors and reverse causality, making it difficult to accurately reveal the true association pattern between hypertension and RDW.^[15]^

This study aims to systematically explore whether hypertension has a causal elevating effect on RDW through MR-PRESSO-adjusted Mendelian randomization (MR) method combined with nonlinear association analysis of data from the National Health and Nutrition Examination Survey (NHANES) 2021 to 2023, and clarify the association pattern between them, so as to provide a scientific basis for in-depth understanding of the pathophysiological mechanism of hypertension and finding new targets for the prevention and treatment of cardiovascular diseases.

## 2. Methods

### 2.1. NHANES

#### 2.1.1. Data source and selection of research subjects

**Figure 1.**
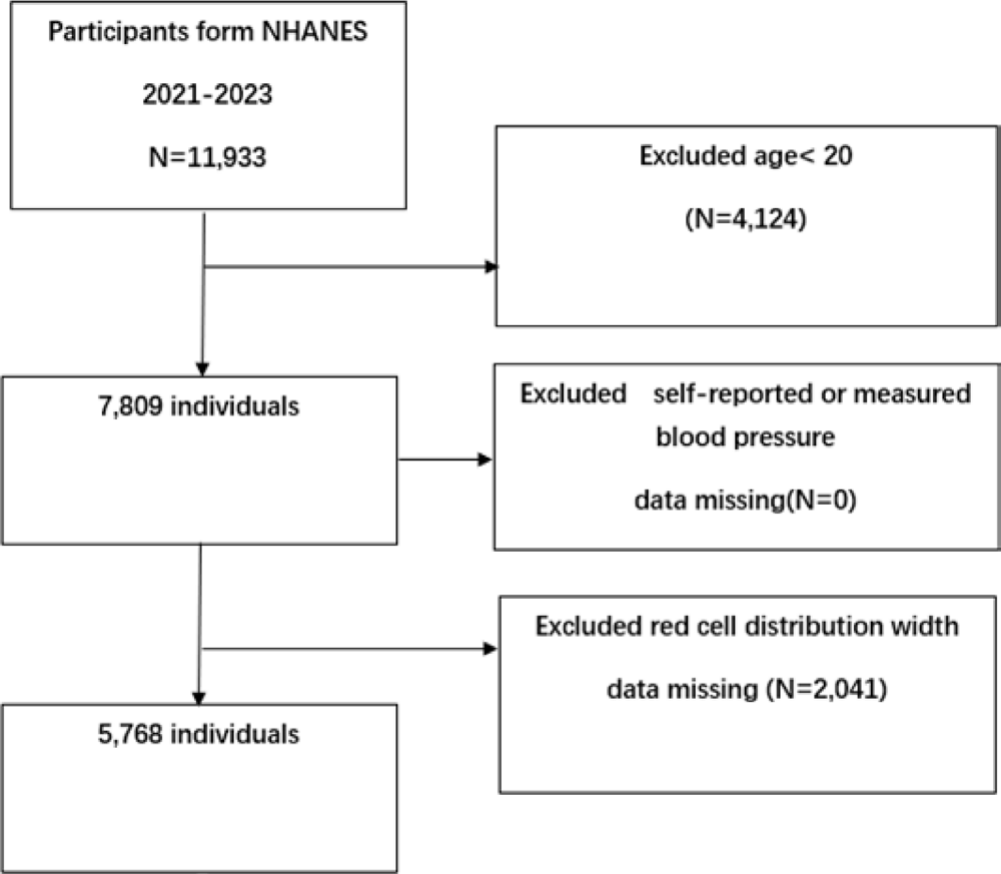
Sample selection flowchart of NHANES 2021 to 2023. NHANES = National Health and Nutrition Examination Survey.

The data used in this study were sourced from the NHANES conducted from 2021 to 2023. The research protocol has received ethical approval from the Research Ethics Review Committee of the National Center for Health Statistics (NCHS). Before data collection, written informed consent forms were obtained from all adult participants and the legal authorized representatives of minors. Comprehensive documentation regarding the survey methods and ethical considerations can be accessed through the official website https://wwwn.cdc.gov/nchs/nhanes/continuousnhanes/default.aspx?Cycle=2021-2023. Initially, there were 11,933 participants. The exclusion criteria are as follows: individuals under the age of 20 (4124 individuals); individuals with missing RDW values (2041 individuals); individuals with missing hypertension data: 0. After applying these exclusion conditions, the final analysis sample group included 5768 eligible participants (Fig. 1).

#### 2.1.2. Exposure and outcome variables

Red cell distribution width (RDW) data were obtained from the complete blood cell count (CBC) tests within the NHANES 2021 to 2023 database. RDW was measured using impedance-based hematology analyzers (Beckman Coulter DxH 800 series, as specified by NHANES). The parameter is calculated as the coefficient of variation of red blood cell volume, expressed as a percentage, using the formula: RDW (%) = (standard deviation of red cell volume/mean corpuscular volume) × 100%.

In this study, the diagnosis of hypertension cases was based on the following criteria: participants who answered “yes” to the question “Have you ever been informed by a doctor or health professional that you have hypertension?”; participants with an average systolic blood pressure >140 mm Hg or an average diastolic blood pressure >90 mm Hg; patients who were currently receiving oral antihypertensive medication. According to these criteria, a total of 5768 patients were finally included in this study.^[16]^

Based on existing literature and data, this study collected potential covariates related to red blood cell distribution width and hypertension, including age, gender, ratio of family income to poverty, educational level, alcohol consumption, high cholesterol level, diabetes mellitus, congestive heart failure, coronary heart disease, stroke, smoking status.

The ratio of family income to poverty was calculated by dividing total annual family (or individual) income by the poverty guidelines specific to the survey year.

High cholesterol level: information is obtained through face-to-face interviews, with the interview question being “Has a doctor or health professional ever told you that you have high cholesterol level?” If the participant answers “Yes,” it is determined that there is high cholesterol level.

Diabetes: the assessment is based on the “self-report diagnosis + history of hypoglycemic drug use” dual criteria. Firstly, the diagnostic information is collected through the interview question “Has a doctor or health professional ever informed you that you have diabetes (including type 1 or type 2 diabetes)?” Those who answer “Yes” are initially determined to have diabetes; Secondly, if the participant reports currently taking hypoglycemic drugs (such as metformin, insulin, etc), regardless of whether they self-reported a diabetes diagnosis or the results of their blood glucose tests, they are directly determined to have diabetes. Participants with gestational diabetes in this study are not included in the statistical category.

Congestive heart failure: it is evaluated through a specific interview, the question being “Has a doctor or health professional explicitly informed you that you have congestive heart failure?” If the participant answers “Yes,” it is determined that there is congestive heart failure.

Coronary heart disease: the interview question is “Have a doctor or health professional clearly informed you that you have coronary heart disease?” Participants who answered “Yes” were determined to have coronary heart disease.

Stroke: this was assessed through the question “Have doctors or health professionals clearly informed you that you have had a stroke?” Participants who answered “Yes” were determined to have a history of stroke.

Smoking: based on the participants’ self-report of their current smoking behavior, the interview question was “Smoked at least 100 cigarettes in life?” If the participant answers “Yes,” it is determined that they have engaged in smoking behavior.

Alcohol consumption: the interview question is “Ever had a drink of any kind of alcohol?” If a participant answers “Yes,” it is determined that they have engaged in alcohol consumption behavior.

#### 2.1.3. Statistical analysis

Statistical analyses were performed using R software (version 4.5.1; R Foundation for Statistical Computing, Vienna, Austria), with inclusion of NHANES complex sampling weights to account for the stratified multi-stage probability sampling design. The statistical significance level was set at 2-tailed α = 0.05. Continuous variables were compared using Student *t* test adjusted for complex sampling design, and categorical variables were compared using Pearson Chi-square test adjusted for complex sampling design.

Considering the complex survey design (stratification, clustering, and weighting), 3 multivariate logistic regression models were constructed to evaluate the association between red blood cell distribution width (RDW) and the risk of hypertension: model 1 (unadjusted); model 2 (adjusted for age, gender, and ratio of family income to poverty line); model 3 (adjusted for age, gender, ratio of family income to poverty line, educational level, alcohol consumption, high cholesterol level, diabetes mellitus, congestive heart failure, coronary heart disease, stroke, and smoking status). Results are presented as odds ratios (ORs) with their 95% confidence intervals (CIs).

Restricted cubic splines (RCS) were used to explore the nonlinear relationship between RDW and hypertension. Stratified analyses were conducted for the complex survey design, and interaction tests were performed to assess potential effect modification across different subgroups.

### 2.2. Mendelian randomization

#### 2.2.1. Mendelian randomization analysis design and data sources

**Figure 2.**
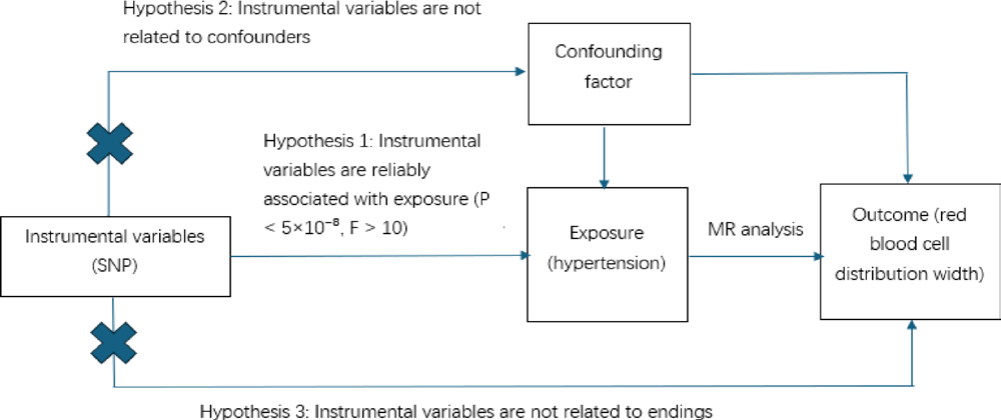
Diagram of Mendelian randomization analysis. SNP = single nucleotide polymorphisms.

In this study, we obtained relevant data from GWAS databases, and Figure 2 shows a schematic diagram of the study design. To investigate the causal association between hypertension and red blood cell distribution width (RDW), we conducted a MR analysis. The core assumptions of MR analysis are as follows: the selected genetic variants [serving as instrumental variables (IVs)] are strongly associated with hypertension (the exposure); these genetic variants are not associated with confounding factors that may confound the association between hypertension and RDW; these genetic variants affect RDW (the outcome) only through their influence on hypertension, rather than through other independent pathways (Fig. 1). Specifically: the GWAS data in this study were obtained from the public database https://gwas.mrcieu.ac.uk/, where the hypertension-related dataset is ukb-b-12493 and the RDW-related dataset is ebi-a-GCST006804, both of which are based on European populations. Since this study is a secondary analysis of public data, no additional ethical approval or informed consent is required.

#### 2.2.2. Statistical analysis

This study employed MR analysis to investigate the potential causal relationship between hypertension (IVs derived from GWAS ID: ukb-b-12493) and red blood cell distribution width (RDW) (IVs derived from GWAS ID: ebi-a-GCST006804). All statistical analyses were performed using R software (version 4.5.1). Initially, single nucleotide polymorphisms (SNPs) significantly associated with the exposure (hypertension) (*P* < 5 × 10^−8^) were selected as instrumental variables (IVs) from the respective GWAS databases. Subsequently, linkage disequilibrium clumping was performed on these SNPs using the European population reference panel from the 1000 Genomes Project (1KG EUR) (parameters: clump_kb = 10000, clump_r2 = 0.001, clump_p = 5 × 10^−8^). The *F*-statistic was calculated for each SNP; only SNPs with individual *F* > 10 and an average *F* > 10 were retained to ensure sufficient instrument strength. Next, MR-Pleiotropy Residual Sum and Outlier (MR-PRESSO) analysis was applied to detect and remove outliers. The primary causal effect estimate was initially assessed using the inverse-variance weighted (IVW) method. Accompanying tests included: Cochran *Q* test for heterogeneity under the IVW framework (*P* < .05 indicating the necessity for a random-effects model), and the MR-Egger intercept test for directional pleiotropy (*P* < .05 suggesting the presence of directional pleiotropy). The final causal effect estimate was primarily derived using the random-effects IVW method, supplemented by sensitivity analyses employing the weighted median, MR-Egger, and weighted mode methods. Finally, a leave-one-out analysis was conducted to verify that the results were not unduly driven by any single SNP.

## 3. Result

### 3.1. NHANES study results

#### 3.1.1. Baseline characteristics of the study population

Table 1 presents the survey-weighted baseline characteristics of the 5768 participants from the NHANES 2021 to 2023 cycle, stratified by hypertension status. Compared to individuals without hypertension (n = 3199), those with hypertension (n = 2569) demonstrated significantly higher red cell distribution width (RDW) (14.0% [95% CI = 14.0–14.1] vs 13.6% [95% CI = 13.6–13.7], *P* < .001), were considerably older (58.9 years [95% CI = 57.9–59.9] vs 43.1 years [95% CI = 42.0–44.2], *P* < .001), and had a lower ratio of family income to poverty (2.9 [95% CI = 2.8–3.1] vs 3.1 [95% CI = 2.9–3.3], *P* = .0147). Significant disparities were also observed in education level (*P* < .001), with a higher proportion of hypertensive individuals having less than a high school education and a lower proportion being more than high school. Hypertensive participants exhibited markedly higher prevalences of high cholesterol level (55.6% [95% CI = 52.8–58.4] vs 24.0% [95% CI = 21.8–26.5], *P* < .001), diabetes (22.0% [95% CI = 19.7–24.6] vs 5.4% [95% CI = 4.4–6.5], *P* < .001), congestive heart failure (6.6% [95% CI = 5.5–7.9] vs 0.7% [95% CI = 0.4–1.2], *P* < .001), coronary heart disease (8.3% [95% CI = 7.0–9.7] vs 1.7% [95% CI = 1.2–2.3], *P* < .001), stroke (6.8% [95% CI = 5.7–8.1] vs 1.5% [95% CI = 1.1–1.9], *P* < .001), and current smoking (44.7% [95% CI = 40.4–49.0] vs 33.6% [95% CI = 29.7–37.6], *P* < .001). No statistically significant differences were found between groups regarding gender distribution (*P* = .2968) or alcohol consumption status (*P* = .116). Data are presented as survey-weighted mean (95% CI) for continuous variables and survey-weighted percentage (95% CI) for categorical variables (Table 1).

**Table 1:** Weighted participants demographics and baseline characteristics.

Characteristic	Hypertension (%)			*P*-value
	Overall (n = 5,768)	No (n = 3,199)	Yes (n = 2,569)	
Red cell distribution width (%)	13.8 (13.7, 13.8)	13.6 (13.6, 13.7)	14.0 (14.0, 14.1)	<.001
Gender (%)				
Male	48.3 (47.0, 49.6)	47.9 (46.1, 49.8)	49.0 (47.6, 50.5)	.2968
Female	51.7 (50.4, 53.0)	52.1 (50.2, 53.9)	51.0 (49.5, 52.4)	
Age (yr)	49.0 (48.0, 50.0)	43.1 (42.0, 44.2)	58.9 (57.9, 59.9)	<.001
Ratio of family income to poverty	3.0 (2.9, 3.2)	3.1 (2.9, 3.3)	2.9 (2.8, 3.1)	.0147
Education level (%)				
Less than high school	35.8 (31.1, 40.8)	32.3 (28.0, 37.0)	41.7 (36.3, 47.3)	<.001
High school	29.5 (26.3, 33.0)	29.0 (25.3, 33.1)	30.3 (27.2, 33.7)	
More than high school	34.7 (28.7, 41.2)	38.7 (32.0, 45.7)	27.9 (23.0, 33.5)	
Alcohol consumption (%)				
No	7.3 (6.1, 8.6)	6.7 (5.5, 8.2)	8.2 (6.5, 10.2)	.116
Yes	92.7 (91.4, 93.9)	93.3 (91.8, 94.5)	91.8 (89.8, 93.5)	
High cholesterol level (%)				
No	64.2 (62.2, 66.1)	76.0 (73.5, 78.2)	44.4 (41.6, 47.2)	<.001
Yes	35.8 (33.9, 37.8)	24.0 (21.8, 26.5)	55.6 (52.8, 58.4)	
Diabetes (%)				
No	88.4 (86.8, 89.8)	94.6 (93.5, 95.6)	78.0 (75.4, 80.3)	<.001
Yes	11.6 (10.2, 13.2)	5.4 (4.4, 6.5)	22.0 (19.7, 24.6)	
Congestive heart failure (%)				
No	97.1 (96.3, 97.7)	99.3 (98.8, 99.6)	93.4 (92.1, 94.5)	<.001
Yes	2.9 (2.3, 3.7)	0.7 (0.4, 1.2)	6.6 (5.5, 7.9)	
Coronary heart disease (%)				
No	95.9 (95.1, 96.5)	98.3 (97.7, 98.8)	91.7 (90.3, 93.0)	<.001
Yes	4.1 (3.5, 4.9)	1.7 (1.2, 2.3)	8.3 (7.0, 9.7)	
Stroke (%)				
No	96.5 (96.0, 97.0)	98.5 (98.1, 98.9)	93.2 (91.9, 94.3)	<.001
Yes	3.5 (3.0, 4.0)	1.5 (1.1, 1.9)	6.8 (5.7, 8.1)	
Smoking (%)				
No	62.3 (58.4, 66.0)	66.4 (62.4, 70.3)	55.3 (51.0, 59.6)	<.001
Yes	37.7 (34.0, 41.6)	33.6 (29.7, 37.6)	44.7 (40.4, 49.0)	

Data in the table: for continuous variables: survey-weighted mean (95% CI).

For categorical variables: survey-weighted percentage (95% CI).

Diabetes (%) (refers to type 1 and type 2 diabetes, excluding gestational diabetes).

#### 3.1.2. Multivariate logistic regression analysis of RDW and the risk of hypertension

Table 2 presents the survey-weighted multivariable logistic regression analyses examining the association between red cell distribution width (RDW) and hypertension risk. In the unadjusted model (model 1), each unit increase in continuous RDW was significantly associated with a 27% higher odds of hypertension (OR 1.27, 95% CI 1.18–1.38, *P* < .001). When RDW was categorized into quartiles (Q1 as reference), participants in Q2 (OR 1.73, 95% CI 1.45–2.06, *P* < .001) and Q3 (OR 2.76, 95% CI 2.33–3.26, *P* < .001) exhibited significantly elevated hypertension risk compared to Q1, with a strong positive dose-response relationship (P for trend < 0.001). After survey-weighted adjustment for age, gender, and the ratio of family income to poverty (model 2), the association remained significant for continuous RDW (OR 1.17, 95% CI 1.10–1.24, *P* < .001), Q2 (OR 1.23, 95% CI 1.07–1.42, *P* < .05), Q3 (OR 1.67, 95% CI 1.36–2.06, *P* < .001), and the trend (*P* < .001). Further survey-weighted adjustment for education level, alcohol consumption, high cholesterol level, diabetes, congestive heart failure, coronary heart disease, stroke, and smoking (model 3) attenuated the associations but they remained statistically significant for continuous RDW (OR 1.13, 95% CI 1.05–1.21, *P* < .05), Q2 (OR 1.21, 95% CI 1.03–1.42, *P* < .05), Q3 (OR 1.54, 95% CI 1.20–1.97, *P* < .05), and the trend (*P* < .001). These results indicate a robust, independent positive association between higher RDW levels and increased hypertension risk, persisting after comprehensive adjustment for covariates and accounting for the complex sampling design of NHANES.

**Table 2:** Association between red cell distribution width and hypertension risk.

RDW	Model 1		Model 2		Model 3	
	OR (95%)	*P*	OR (95%)	*P*	OR (95%)	*P*
Continuous	1.27 (1.18, 1.38)	<.001	1.17 (1.10, 1.24)	<.001	1.13 (1.05, 1.21)	<.05
Tertiles						
Q1 (n = 1654)	Reference	–	Reference	–	Reference	–
Q2 (n = 2085)	1.73 (1.45, 2.06)	<.001	1.23 (1.07, 1.42)	<.05	1.21 (1.03, 1.42)	<.05
Q3 (n = 2029)	2.76 (2.33, 3.26)	<.001	1.67 (1.36, 2.06)	<.001	1.54 (1.20, 1.97)	<.05
*P* for trend	<.001	–	<.001	–	<.001	–

Model 1: unadjusted; Model 2: adjusted for age, gender, and ratio of family income to poverty; Model 3: further adjusted for education level, alcohol consumption, high cholesterol level, diabetes, congestive heart failure, coronary heart disease, stroke, smoking.

#### 3.1.3. The non-linear relationship between RDW and the risk of hypertension and the analysis of threshold effects

**Figure 3.**
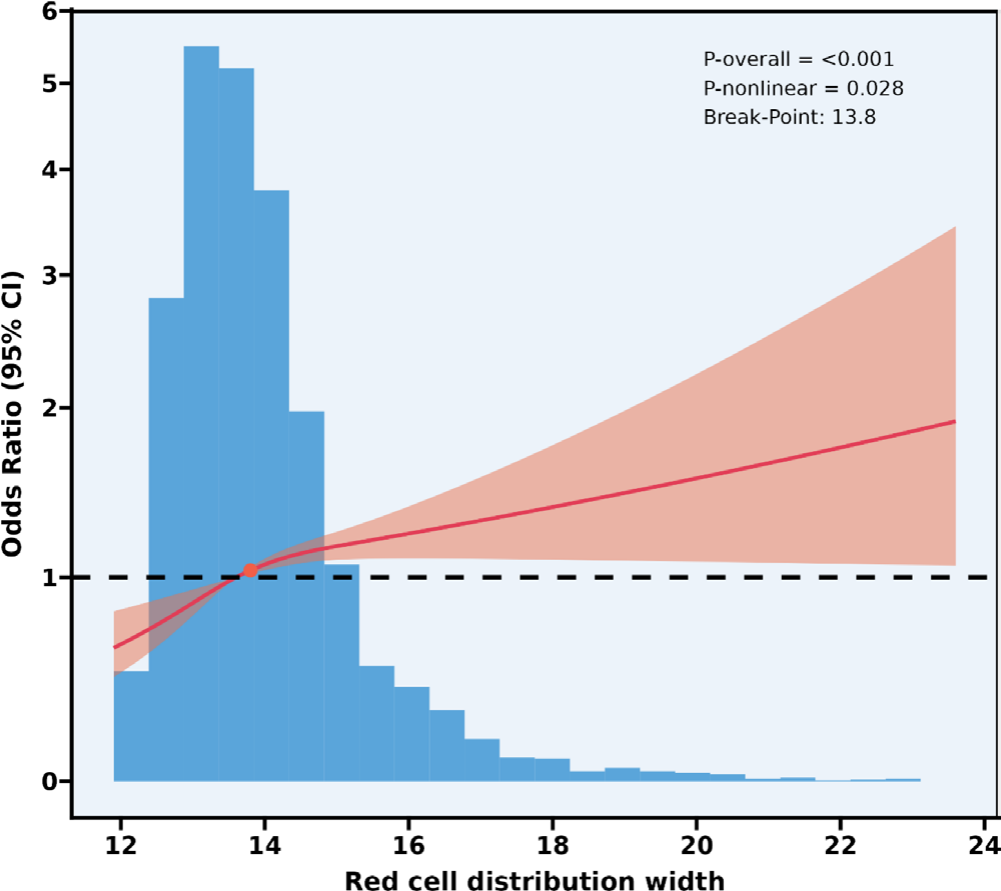
Nonlinear association between red cell distribution width and hypertension risk. Adjusted for age, gender, and ratio of family income to poverty, education level, alcohol consumption, high cholesterol level, diabetes, congestive heart failure, coronary heart disease, stroke, smoking. CI = confidence interval.

Figure 3 and Table 3 present the results of the nonlinear relationship and threshold effect analysis between red cell distribution width (RDW) and hypertension risk. The restricted cubic spline curve (Fig. 3) revealed a significant nonlinear association (*P*-nonlinear = .028) between RDW and hypertension risk after adjustment for age, gender, ratio of family income to poverty, education level, alcohol consumption, high cholesterol level, diabetes, congestive heart failure, coronary heart disease, stroke, and smoking. Visual inspection suggested a potential change point around RDW = 13.8%. Piecewise linear regression analysis confirmed this threshold effect (log-likelihood ratio test *P* = .035). Below the inflection point (RDW < 13.8%), each unit increase in RDW was associated with a substantially higher risk of hypertension (OR 1.29, 95% CI 1.11–1.50, *P* < .001). Above the threshold (RDW ≥ 13.8%), the association persisted but was markedly attenuated (OR 1.07, 95% CI 1.01–1.13, *P* = .031). The overall association remained highly significant (*P*-overall < .001).

**Table 3:** threshold effect analysis of red cell distribution width on hypertension.

Fitting by piecewise logistic regression model (break-point = 13.8)	OR (95% CI)*	*P*-value
Red cell distribution width < 13.8	1.29 (1.11, 1.50)	<.001
Red cell distribution width ≥ 13.8	1.07 (1.01, 1.13)	.031
Log-likelihood ratio	–	.035

Adjusted for age, gender, and ratio of family income to poverty, education level, alcohol consumption, high cholesterol level, diabetes, congestive heart failure, coronary heart disease, stroke, smoking.

#### 3.1.4. Subgroup analysis and interaction test of the association between RDW and hypertension

Table 4 presents subgroup analyses incorporating NHANES 2021 to 2023 complex sampling weights. After adjusting for age, gender, income-to-poverty ratio, education, alcohol consumption, high cholesterol level, diabetes, congestive heart failure, coronary heart disease, and stroke, RDW consistently demonstrated positive associations with hypertension. No significant effect modifications were observed in subgroups of gender (*P*-interaction = .5659), diabetes (*P*-interaction = .8685), high cholesterol level (*P*-interaction = .8337), or heart failure (*P*-interaction = .1476). Strikingly, significant effect modifications emerged for stroke history and coronary heart disease (*P*-interaction < .05): Elevated RDW increased hypertension risk in stroke-free individuals (OR = 1.14, 95% CI = 1.07–1.21) but not in those with prior stroke (OR = 0.93, 95% CI = 0.80–1.09). Similarly, a significant association existed in participants without coronary heart disease (OR = 1.14, 95% CI = 1.07–1.21) but vanished in those with history (OR = 0.87, 95% CI = 0.66–1.13).

**Table 4:** Subgroup analysis for the association between red cell distribution width and hypertension.

Subgroup	N	OR and 95% CI	*P*	*P*-interaction
Gender				
Male	2583	1.16 (1.06, 1.27)	<.05	.5659
Female	3185	1.11 (1.03, 1.21)	<.05	
Diabetes				
Yes	818	1.14 (0.93, 1.40)	.4241	.8685
No	4950	1.12 (1.06, 1.19)	<.05	
High cholesterol level				
Yes	2406	1.12 (1.00 1.24)	.2981	.8337
No	3362	1.13 (1.05, 1.21)	<.05	
Congestive heart failure				
Yes	242	0.97 (0.80, 1.17)	.804	.1476
No	5526	1.13 (1.06, 1.21)	<.05	
Stroke				
Yes	261	0.93 (0.80, 1.09)	.5305	<.05
No	5507	1.14 (1.07, 1.21)	<.05	
Coronary heart disease				
Yes	313	0.87 (0.66, 1.13)	.4883	<.05
No	5455	1.14 (1.07, 1.21)	<.05	

Adjusted for age, gender, and ratio of family income to poverty, education level, alcohol consumption, high cholesterol level, diabetes, congestive heart failure, coronary heart disease, stroke, smoking.

### 3.2. Mendelian randomization results

**Figure 4.**
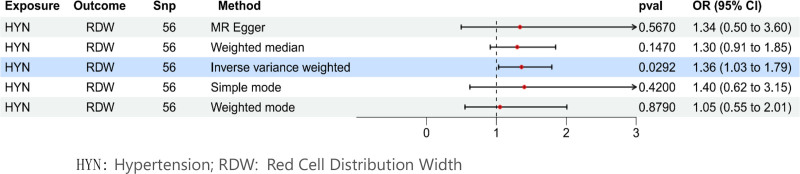
This forest plot shows the relationship between hypertension and RDW in MR. HYN = hypertension, MR = Mendelian randomization, RDW = red cell distribution width.

**Figure 5.**
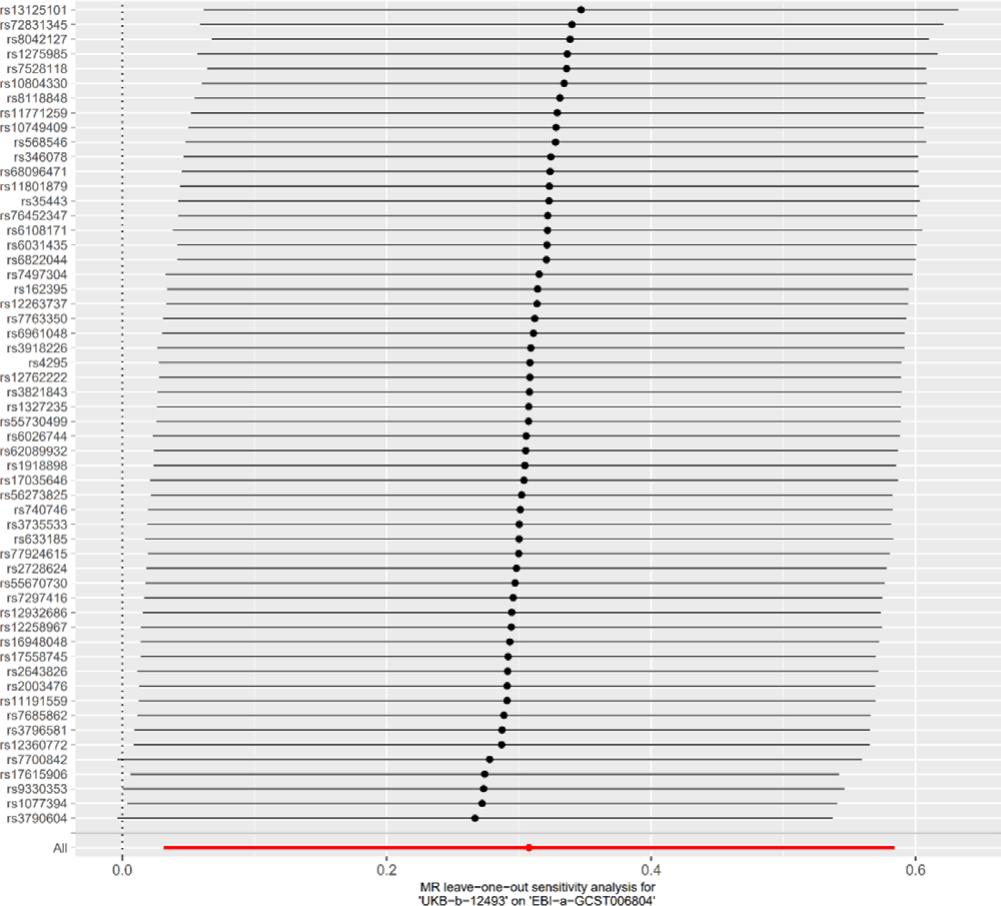
Leave-one-out sensitivity analysis of hypertension on RDW. RDW = red cell distribution width.

**Figure 6.**
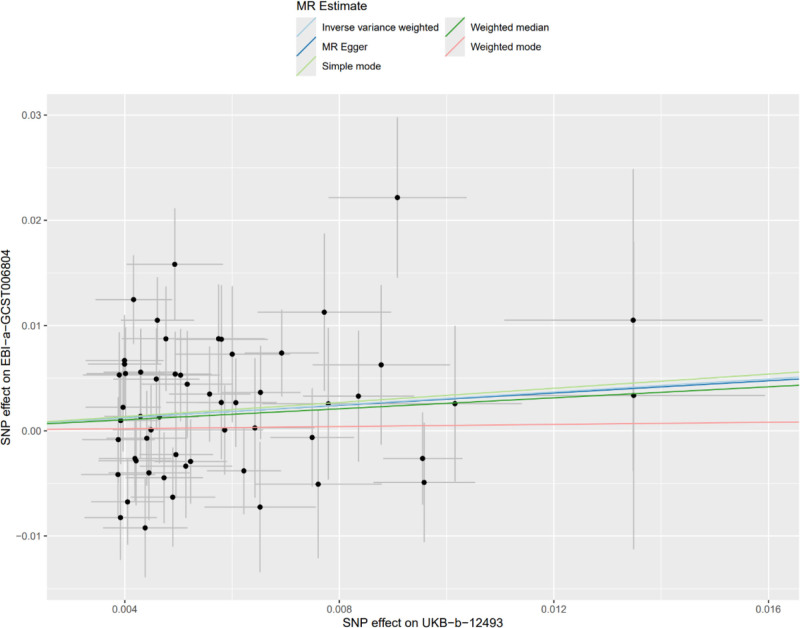
Scatter plot of SNP effects on hypertension (exposure) and RDW (outcome). RDW = red cell distribution width, SNP = single nucleotide polymorphisms.

In the MR analysis with hypertension as the exposure factor and RDW as the outcome factor, after MR-PRESSO correction, 56 SNPs were extracted. The results showed that IVW (OR = 1.36, 95% CI = 1.03–1.79, *P* = .03), MR-Egger (OR = 1.34, 95% CI = 0.50–3.60, *P* = .57), weighted median (OR = 1.30, 95% CI = 0.91–1.85, *P* = .15), simple mode (OR = 1.40, 95% CI = 0.62–3.15, *P* = .42), and weighted mode (OR = 1.05, 95% CI = 0.55–2.01, *P* = .88) were obtained, among which IVW had a significant effect (Fig. 4). Cochran *Q* test indicated heterogeneity between hypertension and RDW (*P* < .05). MR-Egger analysis showed no horizontal pleiotropy between hypertension and RDW, with an intercept of 9.3 × 10^−5^, *P* = .973 (Table 5). The forest plot of the causal relationship between hypertension and RDW is shown in Figure 5, when any one of the 56 IVs (SNPs) was excluded one by one, the estimated causal effect of hypertension on RDW did not show any significant deviation, and remained within the confidence interval. There was no situation where the overall effect direction or significance changed due to the exclusion of a single SNP. This suggests that the results of the MR analysis in this study have good stability and there is no phenomenon where a single SNP dominates the overall causal effect. It further verifies the reliability of the conclusion that hypertension has a causal effect on RDW. The scatter plot of SNP expression for hypertension and RDW is shown in Figure 6. The total sample size for hypertension was 463,010, and that for RDW was 116,666, with *F*-statistic values all >10.

**Table 5:** Sensitivity analysis.

Cochran *Q* test		*Q*	*Q*_*df*	*Q*_pval
Exposure HYN	Outcome RDW	82.585	55	0.009
MR-Egger pleiotropy test	–	Intercept	se	pval
Exposure HYN	Outcome RDW	9.3 × 10⁻⁵	0.003	0.973

HYN = hypertension, RDW = red cell distribution width.

## 4. Discussion

This study innovatively combined the observational analysis of the NHANES from 2021 to 2023 with the MR study corrected by MR-PRESSO, systematically exploring the relationship between hypertension and red blood cell distribution width (RDW). The main research results are as follows: after correction by MR-PRESSO, it was confirmed that hypertension has a causal effect on increasing RDW (IVW method: OR = 1.36, 95% CI = 1.03–1.79, *P* = .03); there is a significant nonlinear association between RDW and the risk of hypertension, with a threshold effect of 13.8%; the association between RDW and hypertension is significant in the population without stroke or coronary heart disease history, but disappears in the population with these diseases. These results provide new insights into the pathophysiological association between hypertension and RDW.

Although previous epidemiological studies have reported the correlation between hypertension and RDW.^[17]^ For instance, a cross-sectional study involving 44,070 adult participants in the United States showed that RDW was positively correlated with the prevalence of hypertension,^[18]^ the causal direction has always remained unclear due to confounding factors (such as age, inflammation) and the existence of reverse causality. This study overcame these limitations through MR analysis and after correction by MR-PRESSO to eliminate outliers and horizontal pleiotropy (Egger intercept = 9.3 × 10^−5^, *P* = .973), the IVW method confirmed that hypertension can causally increase RDW. This finding indicates that hypertension is not only associated with an increase in RDW, but also plays an active role in driving the increase in RDW. The potential mechanisms may include: oxidative stress and chronic inflammation: hypertension induces vascular endothelial damage, promoting pro-inflammatory cytokines (such as TNF-α, IL-6) and reactive oxygen species release. These factors interfere with bone marrow red blood cell production or shorten red blood cell lifespan, leading to an increase in RDW.^[19–21]^ Hemodynamic changes: persistent hypertension may damage the microcirculation perfusion of the bone marrow, affecting red blood cell maturation, resulting in uneven size distribution of red blood cells.^[22]^

The restricted cubic spline (RCS) analysis shows that there is a nonlinear relationship between RDW and the risk of hypertension (nonlinear *P* = .028), with a threshold of 13.8%. Below this threshold (RDW < 13.8%), for every 1-unit increase in RDW, the risk of hypertension increases by 29% (OR = 1.29, *P* < .001); while above the threshold, the association weakens (OR = 1.07, *P* = .031). This threshold effect has important clinical significance, suggesting that when RDW is <13.8%, RDW is a more sensitive marker for the risk of hypertension. Once RDW exceeds 13.8%, other pathological factors (such as advanced age, combined anemia or severe chronic diseases) may dominate the increase in RDW, weakening its specificity association with hypertension. This also explains why previous studies assuming a linear relationship have contradictory conclusions.^[23,24]^

Subgroup analysis shows that the association between RDW and hypertension is significant in the population without stroke (OR = 1.14) or coronary heart disease (OR = 1.14), but disappears in the population with these diseases (interaction *P* < .05). The reasons may be: pathological overlap: stroke, coronary heart disease share common risk factors (such as atherosclerosis), and can independently increase RDW through mechanisms such as chronic ischemia or systemic inflammation. In these populations, an increase in RDW may reflect the severity of cardiovascular complications rather than hypertension itself, thereby masking the specific association.^[25,26]^ Disease progression: in patients with already developed cardiovascular complications, hypertension may be more severe, and its impact on RDW may be masked by other pathological processes (such as anemia caused by heart failure or renal dysfunction).^[27,28]^

### 4.1. Advantages and limitations

The advantages of this study lie in: the mixed design (observational analysis + MR) enhances the causal inference ability and resolves confounding and reverse causality issues. By using the complex sampling weights of NHANES and adjusting 12 covariates, the robustness and extrapolation of the observational results are strengthened. The RCS and threshold effect analysis reveal nonlinear relationships, deepening the understanding of the association between RDW and hypertension. The MR-PRESSO and various sensitivity analyses (such as MR-Egger, weighted median) ensure the reliability of causal conclusions by reducing the influence of multiple effects and outliers.

The limitations of this study are that RDW is a nonspecific indicator affected by multiple factors (such as iron deficiency, vitamin B12 deficiency, hemolysis),^[29,30]^ and these factors were not fully controlled in this study. The sample size of subgroup analysis (such as stroke or coronary heart disease patients) is small, which may reduce the statistical power. The cross-sectional design of NHANES cannot capture the dynamic changes of RDW and hypertension over time. The *Q* value of heterogeneity test *P* < .05 may affect the accuracy of effect estimation, and future more rigorous prospective cohort studies or randomized controlled trials are needed to determine the relationship between hypertension and RDW, minimize the interference of confounding factors, and further verify the reliability of the results of this study.

## 5. Conclusion

This study is the first to explore the association between hypertension and RDW via MR-PRESSO-adjusted MR, providing evidence of a potential causal link where hypertension may contribute to increased RDW, and identifies a RDW threshold of 13.8%. RDW can serve as a prognostic marker for cardiovascular events and may be a reference indicator for monitoring the risk of hypertension in populations without cardiovascular complications, pending further validation of its role in the pathological mechanism of hypertension.

## Acknowledgments

A special thanks to all of the NHANES participants who freely gave their time to make this and other studies possible.

## Author contributions

**Conceptualization:** Zihao Zhao.

**Data curation:** Zihao Zhao.

**Formal analysis:** Zihao Zhao.

**Funding acquisition:** Zihao Zhao.

**Investigation:** Zihao Zhao, Weizhong Huangfu.

**Methodology:** Zihao Zhao.

**Project administration:** Zihao Zhao.

**Resources:** Zihao Zhao.

**Software:** Zihao Zhao, Yuhong Ma, Weizhong Huangfu.

**Supervision:** Yuhong Ma, Weizhong Huangfu.

**Validation:** Zihao Zhao.

**Visualization:** Zihao Zhao, Weizhong Huangfu.

**Writing – original draft:** Zihao Zhao.

**Writing – review & editing:** Zihao Zhao.
